# “What Can We Do?”: A Psychological Perspective on (Mal)Adaptive Coping Strategies and Barriers to Coping in an Area of Severe Climate Vulnerability in Bangladesh

**DOI:** 10.1007/s12529-024-10329-8

**Published:** 2024-11-26

**Authors:** Kyra Lilier, Michael Mikulewicz, Samiya A. Selim, Syed Tauheed Raihan, Rafia Islam, Jennifer Das, Ina Danquah, Till Bärnighausen, Rainer Sauerborn, Kate Bärnighausen

**Affiliations:** 1 Heidelberg Institute of Global Health (HIGH), Faculty of Medicine and University Hospital, Heidelberg University, Heidelberg, Germany; 2 Department of Environmental Studies, SUNY College of Environmental Science & Forestry, Syracuse, NY USA; 3 Centre for Sustainable Development, University of Liberal Arts, Dhaka, Bangladesh

**Keywords:** Coping, Adaptation, Barriers to coping, Qualitative methods, Climate change

## Abstract

**Background:**

As the mental well-being of billions of people is at risk due to the impacts of climate change, more research is required to better understand the psychological implications of climate vulnerability. This research aims to describe the coping strategies of a climate change affected population and the consequences of adaptation behavior.

**Method:**

We conducted 60 qualitative in-depth interviews to elicit the lived experiences of climate-vulnerable men and women in Bhola, Bangladesh. Interviews were analyzed following the tenets of Grounded Theory.

**Results:**

Identified coping strategies included “resignation” or “help-seeking” as well as barriers to coping, such as limited “efficacy,” limited “time,” or “stigma,” which constrained participants — especially women — in their coping behavior.

**Conclusion:**

Our results indicate that certain barriers can lead people to pursue more easily accessible coping strategies, some of which can be interpreted as maladaptive. It is therefore recommended to lift barriers to coping through community-led interventions, such as platforms for sharing problems and knowledge regarding coping strategies.

**Supplementary Information:**

The online version contains supplementary material available at 10.1007/s12529-024-10329-8.

## Introduction

With the number of people vulnerable to climate change surpassing 3 billion, understanding their health needs is essential for future adaptation policy and planning [1]. While much research looks at securing better physical health, less information is available on supporting the mental well-being of those who experience climate hazards [2–4].

In climate change sciences, adaptation is a key strategy for protecting human health and building climate-resilient communities [5]. However, this future-oriented process of adjusting and learning depends critically on how successful populations cope with past or current stressors. On the other hand, maladaptive coping, which is defined here as the unsustainable use of resources, can impede future adaptation efforts that may depend on these resources in the years and decades to come.

Due to its residents’ relatively high vulnerability to the impacts of climate change [6], including its adverse health effects [7], Bangladesh has become a place of extensive research on climate adaptation and coping [8–11]. Bangladesh is often described as an “adaptation laboratory” [8] providing valuable insights and strategies for other parts of the world [8].

However, within the climate change literature, coping is rarely approached psychologically as a reaction to (general) stress but rather as “non-psychological” coping [12]. This means that coping research in the climate adaptation field has focused on specific coping strategies, or ways of coping (such as financial or food-related coping) [13, 14], coping in specific stress situations (such as the aftermath of natural hazards, like storms or floods) [15], coping with ill-health [16–20], coping on household and community levels [13, 15, 21], or coping of specific sectors such as agriculture or fishing [22, 23]. Psychologically, coping is “the ongoing behavioral, cognitive, and emotional [unconscious or conscious [24]] process of managing stress and the negative effects – biological, psychological, and social – stress can have on people’s lives” [25]. This definition does not include an evaluation of whether coping efforts have healthy or unhealthy outcomes, but refers to the (neutral) psychological process of handling stress. However, healthy or adaptive coping is essential for human health and well-being as stress is an inevitable companion of human life [25]. In other words, psychological coping affects both physical and mental health by shaping (either positively or negatively) the levels of experienced stress [25–28]. Psychological coping is seen to be crucial for adaptation in psychology as well [29], as it does not only directly impact (mental) health but also shapes adaptive behavior and motivation, with implications for how individuals respond to climate change. More active coping strategies such as problem-solving may foster psychological and non-psychological adaptation efforts to climate change through high motivation to learn and adjust, whereas more passive coping strategies such as resignation may impede adaptive action, leading to adverse health impacts and depleted resources.

However, the psychological consequences of climate change are among the most under-researched health outcomes [2–4, 30, 31], despite it being essential for “climate change science” [12] and for interdisciplinary research and policy [12, 32, 33]. While research in psychology has contributed to understanding coping with climate change and its impacts specifically [34], very few studies examine how psychological coping behavior in response to general, daily-life (not only environmental) stress is shaped in areas where populations also experience climate-related stress [29, 35]. To our knowledge, no study has so far investigated how coping strategies could vary in such contexts. With climate change consequences additionally taxing personal resources in climate-vulnerable populations, understanding their coping strategies from a psychological perspective can provide valuable insights into adaptation efforts and can inform more effective policy and programmatic responses — both psychological and non-psychological — aimed at promoting the adaptive capacity of these populations [29].

This article aims to add a psychological perspective to research on coping in the fields of climate change, adaptation, and health. We emphasize that our psychological approach to coping is not a clinical assessment of mental ill-health, but a qualitative description of how people react to stress in an area of environmental change. We present coping strategies and barriers to coping strategies within a population that is one of the most vulnerable to climate change impacts globally. This article explores psychological coping in general (not associated with a specific stressor) on an individual level and in a descriptive way. It discusses how the identified coping strategies and barriers could influence future adaptation efforts in Bangladesh and other climate-vulnerable settings.

## Methods

### Study Setting and Design

We conducted an exploratory qualitative study using semi-structured in-depth interviews around the city of Char Fasson (42,000 inhabitants, 2011) [36] which is located in Bhola, an island in the Bay of Bengal with approximately 1.7 million inhabitants [37]. The main industries are agriculture and fishing [37]. Muslims are the biggest religious group, making up 96% of the population [37]. Bhola has a high poverty rate, high population density, and a low education rate [37, 38]. The island experiences environmental change mostly due to hydro-meteorological events such as cyclones, river erosion, floods, and heavy rainfall, and also drought, with predictions that these disasters will occur more often and more intensely due to climate change [38–40]. Among other factors, environmental change has fueled out-migration [41]. In the field of adaptation to and coping with climate change consequences in Bhola, psychological perspectives remain to be explored. Other research has shown that Bhola residents have recovered rather successfully after natural hazards as they have been forced to adapt to and cope with hazards for a long time [42]. They have been shown to have developed local coping strategies, and migration — practiced widely in the area — has been described as an adaptation strategy [43]. However, research also shows that the coping capacity of the Bhola population is limited due to the loss of traditional livelihoods caused by the impacts of climate change, such as cyclones, flooding or river erosion [44, 45].

### Ethics

The Ethics Committee of the Medical Faculty of Heidelberg University (S-928/2019) and the Ethics Committee of the University of Liberal Arts Bangladesh (OFR009) approved this study. All participants gave written informed consent before the interviews.

### Study Population and Sampling

We employed maximum variation purposive sampling to ensure that we captured the perspectives of a broad sample across age, gender, relationship and child status, profession, and educational level. Our purposive criteria included adult men and women who consented to participate and lived in Bhola for more than 5 years, and who had experienced an event that they considered to be about changing weather, a changing environment, or something that they described as climate change. We included participants who worked in Bhola and who had moved to and from Bhola for employment and those who said they would leave Bhola at some point in the future. We selected participants that lived in the center of Bhola, and also those who lived near the river. The sample size (*n* = 60) was constructed to reach thematic saturation [46]. As we recruited a broad group of participants using a maximum variation sampling technique, our sample size needed to be large to ensure we could reach redundancy and see saturation of themes [41]. Therefore, alongside budget and logistical practicalities, we limited data collection to 60 participants. We started to see repetition of ideas at around 35 interviews and felt we reached saturation of the key themes we present here at 45. As the study was designed to explore different topics in this climate vulnerable setting, including migration, mental and physical health, perspectives on climate change, and place attachment, the study continued to ensure we had reached sufficient data power regarding the variety of topics. This also allowed us to search for deviant cases to ensure what we were seeing in the data was valid.

### Data Collection

From the 12th to the 17th of October 2020, five Bangladeshi research assistants (RAs) (including the authors STR, RI, JD), two women and three men, conducted and audio-recorded two semi-structured, in-depth, in-person interviews a day. RAs were post-graduate students at the University of Liberal Arts in Dhaka and were fluent in English and Bangla. Before data collection, RAs were trained in qualitative data collection via an online platform, where aspects of qualitative research such as bias and constructivism were discussed, to build reflexivity among the RAs.

The research assistants acted as the primary instrument of data collection which included an active role in designing interview guides, conducting interviews, and interpreting data based on interactions with participants. Our semi-structured interview guides allowed for the RAs in-depth exploration of participants’ experiences. We integrated reflexive practices throughout data collection (such as debriefings and regular meetings) to ensure the credibility and reliability of our findings [47, 48].

Interviews were conducted using standardized instruments including a participant information sheet with COVID-19 safety information, an informed consent form, and an interview guide available in both Bangla and English. Socio-demographic data (including sex, age, employment status, children, relationship status, and religion) as well as reflexive and observational notes made by the RAs were captured on cover sheets. A COVID-19 guideline included safety measures for RAs and interviewees, and RAs were tested for COVID-19 before leaving for and returning from Bhola. Interviews were conducted outside, with both researchers and participants socially distancing and wearing masks. RAs explained the purpose of the study before the interviews and built rapport. Interviews lasted about 30–45 min and participants chose the interview location.

Throughout data collection, the lead (KL) and last author (KB) conducted daily debriefing sessions to triangulate findings and discuss data saturation as defined by Patton [49]. This allowed us to amend the interview guide for the subsequent interviews, refine lines of inquiry [50], and revise instruments during the process of data collection. Broad, open-ended questions regarding lived experiences, health experiences, perceptions of migration, the future, and problems in Bhola were asked, including narrative-building questions to elicit broad personal responses. RAs probed on themes that seemed to be of relevance to the participant or themes identified as recurring themes via debriefings. RAs simultaneously translated and transcribed interviews after data collection [51].

### Data Analysis

Interview transcripts, debriefing notes, and reflexive and observational notes were analyzed following the tenets of Grounded Theory and managed using NVivo [52, 53]. Through open, axial, and selective coding, the principal author (KL) inductively developed a codebook with descriptive and content-based codes. Within many of these codes, she found a strong connotation related to mental health. To further explore this connotation, she developed a mind map that comprised themes around mental health informed by the built codes and her medical background that captured and organized topics related to mental health, including stressors as negative influences on mental health, protectors as positive influencers, and coping strategies. Then via our iterative reading, coding, and allocation of codes to themes, we noticed recurring descriptions of behaviors and strategies participants employed to manage stress. For instance, the act of withdrawing from stressful situations to be alone emerged as a common theme. Through multiple processes of abstraction and interpretation, we examined how the specific actions and reflections of participants aligned with existing literature and theories. After reflexive group discussions with the research team, re-reading the notes and transcripts, and mapping our themes to multiple theoretical concepts (including some we rejected such as adaptation concepts), we felt these behaviors related specifically to the concept of coping strategies. For example, the descriptions of seeking solitude and isolation were connected to the concept of coping, as individuals managed emotional distress by creating a psychological or physical distance from the stressor.

**Fig. 1:**
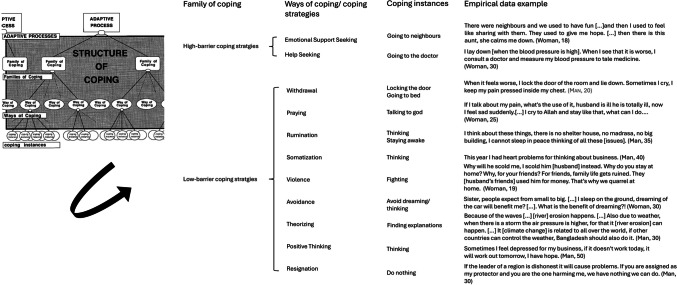
To visualize the thought process behind our analysis and extraction of coping strategies from our empirical data, we display here an adaptation of Skinner’s figure, described as: “A hierarchical conceptualization of the structure of coping. The figure runs off both sides of the page, indicating that there is not a fixed number of adaptive processes, families of coping, ways of coping, or coping instances.” [27] (on the left) with example quotes from the supplemental material. It should be noted that many more instances of coping can be found in the data that are summarized in the coping strategies that we describe. As a key example, the coping strategy of withdrawal summarizes a range of coping instances as the action taken to psychologically withdraw, in this case, “locking the door” and “going to bed”

Inspired by Skinner et al. (2003): “Searching for the Structure of Coping: A Review and Critique of Category Systems for Classifying Ways of Coping” [27], we applied a hierarchical concept of coping when analyzing and conceptualizing the data (Fig. 1). The basis of Skinner’s concept are coping instances, described as “the countless changing real-time responses that individuals use in dealing with specific stressful transactions, such as ‘I wore my lucky socks the day of the surgery’ or ‘I read everything I could find about it.’” [27]. In other words, the specific action taken. Next on the scale, “a set of lower order categories (e.g., problem-solving, rumination, venting, escape) must be identified that can reliably classify instances of coping (observations or items) into conceptually clear, mutually exclusive and exhaustive categories. Often labeled ways of coping or coping strategies, these refer to recognizable action types (Lazarus, 1996).” [27]. These recognizable action types are what we present as coping strategies. Skinner then classifies these coping strategies into families of coping according to their adaptive functions. In “Table 4: Higher Order Distinctions Among Coping Categories” [27], Skinner displays possible higher categories. However, she points out that coping strategies have been conceptualized inconsistently [27, 54] while common classifications include emotion-focused versus problem-focused coping, approach versus avoidance coping or “good” versus “bad” coping [27, 28]. She criticizes many of these categories as “not conceptually clear, mutually exclusive, or exhaustive” [27] and have therefore been proven not useful as classifications [26, 27]. We adapt this hierarchical categorization and, to provide a more useful categorization of families of coping strategies with practical implications, we ordered our coping strategies along the barriers specific to the concept, further described below. This allows us to highlight where the population could be assisted with coping and to open up a context-specific discussion on adaptive versus maladaptive coping behavior.

KL transferred the aforementioned mind map to the codebook to reanalyze the data, where new coping strategies inductively emerged. In the second step, KL reanalyzed all codes that related to coping and mental health whereas she used Skinner’s analysis of coping strategies in “Table 3: Comprehensive List of Lower Order Coping Categories,” as an inspiration in naming and classifying our coping strategies [27], to underpin the identified coping strategies and highlight the less obvious ones. Finally, KL coded barriers to coping that were mentioned within the found coping strategies. KL shared and discussed found narratives and the developed codebook with the lead investigators (SS & KB). Data were coded as coping strategies when participants explained what they did if they felt bad or how they reacted to a stressful situation. Our coping strategies thus refer to the action a participant described after encountering a stressful situation or stressor and the resulting negative feelings. Psychological stressors are defined as “social and physical environmental circumstances that challenge the adaptive capabilities and resources of an organism.” We use this definition to highlight and explain the stressors described by participants.

While some participants also evaluated their strategies as relieving or making them feel better, others reported their strategies without further elaboration. We present coping strategies descriptively, building two groups. The first group includes coping strategies that have been reported without barriers to using them, meaning participants did not mention anything that prevented them from reacting that way (low-barrier coping strategies). The second group comprises coping strategies which participants described to be only accessible under certain conditions (high-barrier coping strategies). Reported barriers are displayed along with the respective coping strategies.

## Results

Coping strategies are presented from an area affected by environmental change and categorized according to their accessibility as high-barrier and low-barrier strategies.

Participants spoke of loss and helplessness, but spoke of developing and using multiple coping strategies, at times simultaneously (some quotes may therefore refer to several strategies). The stressful situations that participants described ranged widely from financial, physical, or environmental stressors to pressure created with and by the social environment. Financial stressors were pervasive, as many participants struggled with insufficient income and lack of access to stable employment opportunities. This financial instability was compounded by physical and environmental stressors, such as the lack of sufficient food, clean water, and safe shelter, which are critical but frequently scarce in this setting. Participants also highlighted the social pressures they faced, including family obligations, family and community expectations, social conventions, or relationship conflicts. The adverse effects of climate and environmental change were described as increasing these stressors. Participants reported that heavy rains, cyclones, droughts, and floods frequently disrupted agricultural production, and also damaged essential infrastructure, such as roads, homes, and irrigation systems. This environmental degradation led to further financial strain, as the costs of limited access to education and healthcare further diminished their capacity to adapt and recover, perpetuating a cycle of stress.

**Fig. 2:**
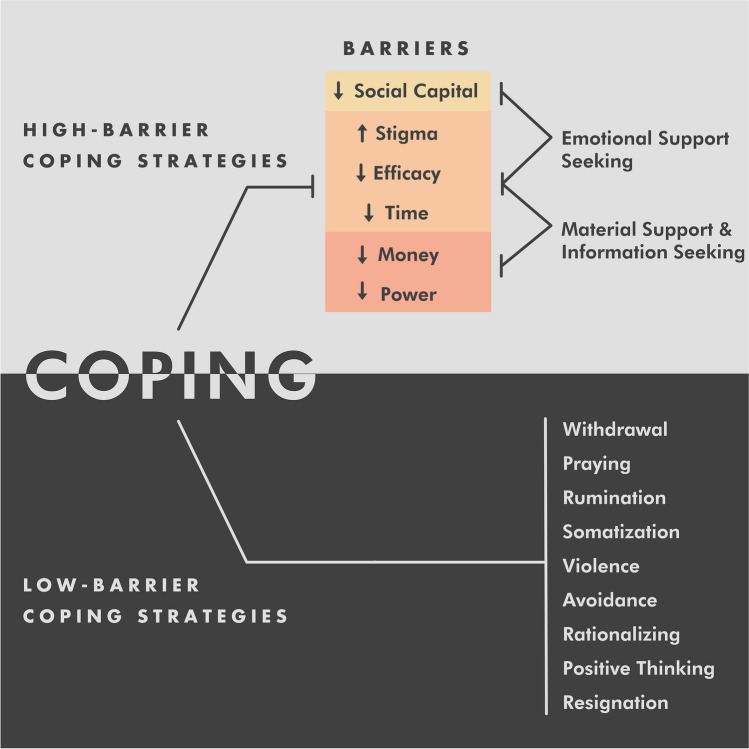
Coping strategies and barriers to coping. Coping strategies are categorized by their accessibility. Low-barrier coping strategies (lower half in dark gray) are strategies with no reported barriers. High-barrier coping strategies (upper half in light gray) are those with many reported barriers. Barriers are presented in the orange box. While high-barrier coping strategies have specific barriers, they also have barriers in common, which are represented by the black lines

The study involved 40 men and 20 women. Participants were between 18 and 85 years old, mostly married, Muslim, and working in the housework or fishing business, and had no formal education (Table 1). It should be noted that women provided deep insights into their coping behavior Fig 2.

**Table 1:** Socio-demographic characteristics of the study population

Characteristic	Number	%
Total	60	100
Female	20	33
Male	40	67
Age group		
18–29 years	22	37
30–49 years	19	32
50–69 years	15	25
70 + years	4	7
Occupation (more than 1 possible)		
Housework	18	30
Fishing	16	27
Farming	9	15
Vending/commerce	8	13
Hotel/restaurant	5	8
Day labor	3	5
Rickshaw driving	2	3
Other	6	10
Religion		
Muslim	58	97
Not disclosed	2	3
Relationship status		
Married	50	83
Single	7	12
Partner, not living together	1	2
Not disclosed	2	3
Number of children		
0 to 2	27	45
3 to 5	21	35
More than 5	12	20
Highest education level		
No formal education	25	42
Primary education	21	35
High school education	7	12
Tertiary education	5	8
Not disclosed	2	3

### Low-Barrier Coping Strategies

In this section, low-barrier coping strategies are presented (e.g., “Withdrawal,” “Avoidance,” or “Resignation”) which participants did not mention any barriers to and which were thus easily accessible.

#### Retreat, Withdrawal, Distracting Oneself, and “Me Time”

Participants described a coping strategy, in which they withdrew from a situation to be by themselves. When doing so, they often cried, lied down and rested, or slept. One participant described crying as relieving (see Supplemental Material, Q1). Withdrawing could also mean that participants engaged in activities to distract themselves. These activities included visiting favorite places, carrying out pleasant activities, or distracting themselves with work (see Supplemental Material, Q2). In some cases, withdrawing meant that participants isolated themselves from their social environment, while in others they concentrated on their inner well-being without necessarily being alone. Withdrawing was often associated with praying, which is discussed separately.

#### Praying

Participants reported resorting to prayer when they were stressed. They explained that they shared their feelings, worries, and hopes with God. They often did so because they felt that there was no one else to share them with and hoped that through praying, God would alleviate their sorrows now or in the afterlife. Praying included both taking time to intentionally say a prayer or speaking some words to God spontaneously (see Supplemental Material, Q3).

#### Rumination

As a reaction to stress, participants described that they ruminated and could not stop thinking about the problem, even if they tried. Some participants noted that they had difficulties sleeping as a result (see Supplemental Material, Q4, Q5).

#### Somatization

Somatization is an unconscious coping strategy, in which the mind reacts to stress through symptoms in the body. While participants did not use the term “somatization,” they explained how their body reacted to stress which led to physical health problems, such as high blood pressure, or skin or heart problems. They reported feeling drained, losing weight, or getting white hair at a young age (see Supplemental Material, Q6, Q7).

#### Violence

Participants reported that being violent — physically and verbally — was a reaction to stress. In most cases, women talked about experiencing violence when their husbands were angry or stressed. They described being beaten or insulted when there was insufficient income, when their husbands had issues at work, or even where there was no obvious reason, at all (see Supplemental Material, Q8). However, in some cases, women said they beat their husbands or started verbal arguments due to tensions regarding family finances (see Supplemental Material, Q9). Violence as a coping strategy was mostly not described by the actors that used this strategy, but by victims or the social environment.

#### Avoidance and Suppression

Participants described blocking out problems, suppressing feelings, and concealing them from others. They expressed pragmatic, avoidant attitudes and suppressed emotion when saying that feelings, dreams, or thinking about the future is not useful. They also explained to have moved on after bad experiences or spoke about distracting themselves, via engaging in social activities (see Supplemental Material, Q10). Additionally, participants reported avoidance coping, a well-known coping strategy in psychology [26]. In particular, they described avoiding social stress by avoiding disagreements or trying not to attract attention in the community. To do so, participants said that they tried to behave according to social conventions when handling social situations, emphasized when they behaved according to social norms, and used excuses when they felt they could not react conventionally. Participants also reacted with shame, concealed problems, or downgraded their importance, especially when they felt their problems were seen as socially unacceptable, e.g., perceived as trivial.

#### Rationalizing

When stressful events occurred, participants tried to find explanations or to assign blame to make sense of them. Very often, they showed fatalistic and superstitious beliefs, for instance when stating that it was fate or God’s will (see Supplemental Material, Q11). They also blamed other people, including fellow community members, the government, or political leaders for being responsible for their problems. In many cases, they shared various theories to explain their situation. Some participants also provided explanations for the environmental change, such as river erosion in the area (see Supplemental Material, Q12).

#### Optimism and Positive Thinking

Participants reported how they reacted to bad situations with optimistic attitudes and positive thinking (see Supplemental Material, Q13). This means that whenever something bad happened to them, they tried to think positively and to see the good side of things, kept hoping for solutions, or reacted with humor. They also mentioned their confidence to overcome problems (see Supplemental Material, Q14).

#### Resignation, Desperation, and Apathy

Many participants expressed resignation when encountering stressful events, often when they felt helpless, hopeless, powerless, or worthless. As a consequence, they would passively endure a stressful situation or give up and do nothing. Some participants explained doing so because they became used to a certain stressful situation over time or because there were no alternatives. Others explained that their fate was in God’s hands, that they did not know how to solve their problems, or that attempts to do had failed (see Supplemental Material, Q15). Resignation could mean that participants stopped acting on a problem but stayed emotionally involved, which often led to feelings of inner emptiness, pessimism, anxiety, or deep desperation. Some participants also talked about regret and showed sarcastic behavior and self-devaluation. Participants also reported withdrawing emotionally and feeling completely resigned, resembling a state of apathy (see Supplemental Material, Q16). When talking about resignation as a coping strategy, a number of participants used phrases like “nothing we can do” or “What can we do”? To emphasize the dominance of these expressions, they and similar phrases were counted, like “nothing (else) I can do” or “what (else) can I do” or “(is there) anything we can do?” within our resignation codes. More than half (31) of the participants used these expressions and they were mentioned more than 80 times in the transcribed data. These phrases were used by a disproportionately high number of women compared to men (70% of women vs. 40% of men). Findings were discussed with RAs (STR, JD, RI) and surmised that these phrases should be understood literally, meaning participants did not know what they could do or did not have any opportunities to act. Overall, “resignation” was the most prominent theme in our data (see Supplemental Material, Q17, Q18).

### High-Barrier Coping Strategies

This section focuses on the high-barrier coping strategies (“Emotional support seeking” and “Material support and information seeking”) which participants described as not easily accessible. Barriers are discussed along with their respective coping strategies.

####  Emotional Support Seeking

As a coping strategy, participants mentioned engaging in social interaction to seek emotional support. They described sharing their feelings or problems with friends, family, neighbors, or people from the community (see Supplemental Material, Q19). Participants were careful about choosing the right person to speak to about intimate topics. For the most part, they felt safer sharing problems and feelings within their families. Seeking emotional support was sometimes accompanied by seeking material help, such as financial support, or goods and products (see Supplemental Material, Q20).

#### Barriers to Emotional Support Seeking

Participants expressed that it was often difficult or impossible for them to engage in social contact to talk about their problems, suggesting low self-efficacy, which is defined as “assessing one’s ability to engage in a behavior” [12]. Some added that there was no use in doing so because it would not lead to any benefit, implying low response efficacy, defined as “the perceived likelihood of a behavior to result in the desired outcome” [12]. Others felt prevented from freely talking about their worries because of social conventions that would lead them to be stigmatized. Participants were ashamed of talking about problems and perceived it as complaining, which is societally unacceptable (see Supplemental Material, Q21). They also described that there was no suitable person to talk to (lack of social capital) or that they feared facing negative consequences, such as defamation or worrying loved ones (see Supplemental Material, Q22). Additionally, participants stated that there was no time to engage in social contact as they had to work all day to sustain their families. Those who had moved in search of jobs explained that the distance separating them from their families was a barrier to receiving emotional support. However, numerous interruptions and comments by neighbors and family members who were well-informed about participants’ worries and problems — observed during a number of interviews — suggest that exchanges about intimate topics do take place. Female participants faced additional barriers to seeking emotional support as they often were not allowed to go out in public to meet at tea stalls or mosques as men do, and had limited opportunities to engage in social contact, in general.

#### Material Support and Information Seeking

Participants identified asking for material support or information seeking as a coping strategy. This also included speaking about their situations and worries in the hope of being heard and getting help. To do so, they often engaged with specific professionals, such as doctors, political leaders, or government members, or asked their neighbors, family, or community members for material support or information on where to get help (see Supplemental Material, Q23). Participants also took loans, accepted government aid, or took shelter in times of bad weather or storms. Seeking help often included assisting others to keep a mutual support system going, in recognition of the mantra that “one good turn deserves another” (see Supplemental Material, Q24).

#### Barriers to Material Support and Information Seeking

Participants reported various barriers to seeking help. Some said they did not have enough time, while others mentioned lack of money as a barrier, for example when trying to access medical treatment. When seeking help from officials or politicians, participants described not having enough power, influence, or education to be able to talk to them (self-efficacy) or enough money to bribe them. Another reported barrier was the feeling that seeking help was not useful as it would not be successful (response-efficacy) (see Supplemental Material, Q25). Social conventions also worked as barriers, as participants explained they were not allowed to complain or ask for help or were ashamed and feared bad consequences if they did so (stigma) (see Supplemental Material, Q26). Though some participants knew about support programs, very few had access to them.

## Discussion

The described coping strategies are from an area of environmental change and are categorized according to their accessibility. The coping strategies themselves, as well as the barriers reported, are not exhaustive or mutually exclusive, or experienced equally by all participants. Similarly, designating coping strategies as “low-barrier” does not necessarily mean that there are no or few barriers to them but rather that none was reported by participants during the interviews. Low-barrier coping strategies comprise “withdrawal,” “praying,” “rumination,” “somatization,” “violence,” “avoidance,” “rationalizing,” “positive thinking,” and “resignation,” while high-barrier coping strategies include “emotional support seeking” and “material support and information seeking.” Reported barriers to these high-barrier coping strategies were limited: “efficacy,” limited “time,” limited “social capital,” limited “power” or “money,” as well as “stigma.”

Bangladesh is particularly affected by climate change consequences, such as adverse weather, changes in weather patterns, precipitation, or heat waves [6]. Hydro-meteorological events such as cyclones are increasing. Cyclone Sidr (2007), Cyclone Aila (2009), and Cyclone Yaas (2021) resulted in high precipitation, floods, and river erosion which disrupted socio-ecological systems [6, 7, 55].

While we do not attribute coping strategies to a specific stressor, our findings reveal a wide range of stressors as described above. Without asking specifically about climate change or using this as a concept within the interview guide, participants spoke of adverse weather events, and coping in relation to this and other existing stressors. For example, participants reacted with resignation or praying to cyclones, as they believe their fate to be in God’s hands.

During cyclones we cannot do anything as our house is here. We were here [during the cyclone] as the center is very far from here. We have rivers here, but nothing to do. The fish [in the pond] were gone, trees were uprooted. Everything went under water, then like a month we suffered. (Man, 50)

People got their trees broken or it takes away the houses and shops, destroy crops, cattle die, […] What to do with this! If God wants to kill us, what can we do? (Man, 37)

Among the wide range of coping strategies used in the face of climate change, participants also applied positive thinking, here in relation to river erosion:

The tide actually breaks everything and we fix everything again. Here, the storm was coming. All taken away. We put soil again. We're all set to stay again. (Woman, 25)

Such examples allowed us to interpret these coping strategies to reflect a holistic perspective of vulnerable populations, where climate is changing and extreme, adverse weather events are increasing.

Few studies look at general psychological coping (not related to a specific stressor, such as a natural disaster) in climate-vulnerable populations, and, to our knowledge, none investigates specific coping strategies within this context [29, 35]. However, research on coping in extreme environments that demand individuals to adapt to survive [56] cites climate change as an additional stressor that can lead to limited coping capacity. Existing research in extreme environments shows overlapping results (e.g., avoidance, seeking help and emotional support, positive thinking, and withdrawal) [56–60]. With regard to limited capacity in extreme environments, Barbarito describes the apathetic immobilization strategy of “freezing” [61], which relates to resignation also observed in our work [61].

Our results also overlap with the strategies used to cope with climate change as a specific stressor in relatively less vulnerable countries such as France [34], where apathy was also observed [62]. Overall, limited coping capacity, and low perceived self-efficacy, seems to lead people to use coping strategies that elicit more passive behavior, have fewer barriers, and focus on the emotional regulation, such as resignation, avoidance, positive thinking, or withdrawal [29, 56, 60, 63]. This is also confirmed by research on coping with poverty-related stress, a stressor also present in our sample, where studies have found that people tend to use coping strategies that are more passive, focused on emotions and avoidant, evasive behavior [64, 65]. However, aligning with our results, people experiencing poverty-related stress also engage in coping strategies that are more active, such as help seeking or social support, or religious coping [66]. These overlapping results from different stressful environments suggest that environmental change does not lead climate change — affected populations to use different coping strategies when adding to ‘normal’ daily-life stress [67]. Rather, it is the amount of experienced stress and uncontrollability (and not the cause of it) that influences coping behavior, in a way that it leads people to use more passive coping strategies. 

The barriers to high-barrier coping strategies are economic (lack of money, time (due to work), political (no power), cultural or societal (stigma), and personal or social (low self-efficacy and lack of social capital) by nature. Importantly, they also intersect on various levels and align with barriers to coping in other settings [4, 68–72]. Regarding lack of social capital, there was a discrepancy in some cases between the participants’ narrative of not being able to share feelings and at the same time interruptions from neighbors who were aware of sensitive personal information. This suggests that some participants did not consider talking to their neighbors about problems and sharing their feelings. Lack of self-efficacy and response efficacy were also reported as barriers. The absence of response efficacy regarding public services has led to feelings of hopelessness in other climate-vulnerable populations, where participants felt they were not supported by government institutions after flooding [73]. Additionally, lacking self-efficacy and low perceived adaptation capacity are thought to impede coping and adaptation by discouraging people from taking action [29, 33].

It is highlighted that the two high-barrier coping strategies of “emotional support seeking” and “material support or information seeking” are part of a complex social system, in which giving and receiving are mutually interlinked. This “one good turn deserves another” philosophy emphasizes that coping should not be understood as a purely individual behavior but as embedded in social relationships, and that an overly individualized approach fails to recognize important aspects of coping [74].

While barriers are presented without further insights on why a barrier is felt, research shows that the socio-economic status (SES), including gender [75], determines how people cope [63, 76–80]. Lower social and material status is linked to lesser availability of material, social, and psychological (like self-esteem, efficacy, and control beliefs) resources, thus impeding people from accessing a wide range of coping strategies [76, 78, 81]. People with lower SES tend to use fewer coping strategies that are active and supportive in favor of the more avoidant, disengaging [78], and maladaptive ones [77]. With gender being a determinant of lower socio-economic status in Bangladesh (this also means that women have less decision-making power and a less self-determined life) [82–85], our study suggests that the socio-cultural implications of gender can be interpreted as an additional barrier to coping. This is reflected in our findings where female participants were less likely to contemplate solutions to their problems due to their social status, aligning with other research on gender inequalities and empowerment [86, 87]. Their disproportionally high use of phrases such as “What can we do?” exemplifies the powerlessness and hopelessness increasingly felt as an additional barrier by women. Therefore, our study suggests that women could be more likely to use low-barrier coping strategies, though further research is required to support this finding. Women were often disallowed to leave their homes, leading to their shrinking ability to cope, as opportunities, e.g., to engage in social contact, were limited for them.

As in other studies, when feeling helpless and hopeless in the face of uncontrollable situations with limited self-efficacy, engaging in religious practices is another coping option [67, 72, 88]. In our study, the coping strategies of “praying” and also “rationalizing” (when participants explained something by stating it was God’s will) depended on believing in God. Religious coping is a common strategy and has been described as both positive and negative for health and well-being [89–93]. It can be a supportive coping strategy, for example when people feel supported by God or reframe adverse events as an opportunity [89–93]. Additionally, it is an easily accessible and culturally embedded strategy [94, 95] that people can engage together in a religious group, which provides them with a sense of belonging and support [80, 90, 92]. However, in our study, the social benefits of engaging in religious coping collectively were less accessible to women, as they often were not allowed to go to mosques on their own. Religious coping can also be maladaptive if people feel punished or abandoned by God [89–93]. This is linked to fatalism [92–94, 96], a belief that a negative event is God’s will and nothing can be done about it, which was often reported in our study and other work from Bangladesh [67, 88, 94]. This attitude could promote a state of resignation and feeling of hopelessness [94] and is associated with clinically diagnosed depression [97].

Whether it is possible to label a specific psychological coping strategy as maladaptive with negative health outcomes or adaptive with positive health outcomes, is unclear [27]. While some studies find that specific coping strategies are healthy or adaptive (such as meaning-making, support-seeking, and emotional expression), others seem to be maladaptive or unhealthy (such as social isolation, rumination, or avoidance) [26, 27, 98]. Some studies find no differences in terms of the health outcomes of coping strategies, at all [99]. Moreover, coping strategies and their outcomes for health are difficult to predict due to being context-specific. This means that in an unchangeable situation, resignation could be healthier than wasting one’s resources [27, 54]. However, while keeping this context-specificity in mind, it is suggested that the prolonged use of coping strategies that decrease resources and increase vulnerability (for example by decreasing self-efficacy) can be termed maladaptive, while coping strategies that help accumulate resources can be termed adaptive [27]. In climate change-affected populations, psychological coping strategies should be evaluated not only by their impact on health, but also by their impact on behavior. For example, resignation — the most frequently used coping strategy in our study — could be useful in handling current, uncontrollable stress with positive mental health implications, but at the same time could decrease self-efficacy and motivation, leading to a state of apathy which — in the long run — could prevent people from learning and exploring new ways to prepare for future stress [29]. Therefore, when considering maladaptive psychological coping strategies, not only adverse (mental) health consequences should be considered, but also behavioral consequences that impact coping with, and adaptation to climate change.

Given the aforementioned insights from the literature, it is reasonable to interpret specific, frequently used coping strategies as more adaptive or maladaptive based on the extent to which they draw upon resources that are necessary for coping and adaptation in the future. Navarro et al. [29] find that avoidance coping and strategies that tend to focus solely on emotional regulation are less adaptive in the long run compared to strategies that tend to lead to actions, such as remaining in a state of vigilance and having a proactive mindset to tackle problems. In our study, the coping strategies of withdrawal, rumination, somatization, violence, avoidance, and resignation could be more maladaptive if frequently used, whereas theorizing, positive thinking, and seeking emotional and material support could be more adaptive. It should be noted that the last two were described as high-barrier coping strategies in our study, meaning they have limited accessibility. This could lead to using more maladaptive, easily accessible coping strategies. It is therefore necessary to create interventions that help overcome those barriers, especially in a setting where climate change is already limiting coping capacity.

The first step for external interventions would be to address the barriers to adaptive coping strategies. To build a coping-enabling environment [74], one has to take into account that coping is not an individual process, but that the social (including political) and natural environment is key [74, 100]. This is reflected in the economic, political, cultural, societal, personal, and social nature of the barriers to coping, which interact on various levels. Thus, multiple or multi-layer interventions are needed to tackle these barriers. It is suggested that successful adaptation is multi-faceted and should be transformational, addressing the root causes of social and climate injustice at different scales [101]. While strengthening social policies, accessibility to education and health services, equity, and fair political representation for all genders and minority groups are indispensable in the long run, front-line community interventions could act as a ‘first aid’ strategy to facilitate coping [102]. A key goal of interventions would be to strengthen self-efficacy and the sense of control over one’s life, as perceived adaptive capacity is crucial and, if positive, increases the motivation to cope [33, 60, 103]. This could be achieved by facilitating the exchange of knowledge and information, thereby creating solutions and opportunities to act. Additionally, the regulation of emotions, the process of adapting to and controlling strong emotions, is an important factor in enabling coping [29]. Other studies showed that self-confidence, support networks, and religious beliefs functioned as facilitators for coping [104] and that high levels of community participation correlate with better mental health [105]. Though some participants mentioned support and relief programs, few of them had access to these. Interventions should encourage and enable people to engage in social contact and share and discuss problems and feelings, while at the same time informing and educating participants about, and guiding them towards, additional support whenever needed.

To achieve this, we recommend community support delivered via trained community workers. These community workers could moderate community-led interventions, such as (gender-specific) group meetings, in order to create a platform for sharing knowledge and problems, informing, educating, and referring attendees for additional support, if needed. This could help raise awareness about common problems and destigmatize negative feelings that are not allowed to be shown, strengthening mental health literacy and lifting fear of stigma. Exchange of knowledge, information, and solutions to problems could strengthen self-efficacy and a sense of control, opening opportunities to act. These interventions could follow the action principles of psychological first aid, which aims to psychologically support people through “looking, listening and linking” (gather information about the situation, ensure safety and basic needs, familiarize actors, active listening, educate and inform, link to social support, guiding to access services) [102]. If stigma prevents people from discussing problems and possible solutions in groups, community workers could also support people through one-on-one meetings. The friendship bench intervention could work as a model [106, 107] on which to develop approaches relevant to the Bangladeshi context. Though this intervention was originally designed to tackle common mental disorders through counseling by a trained community worker on a so-called friendship bench, it could also be opened to people without a (diagnosed) mental health condition as a safe place for sharing problems and knowledge. This could help to share problems more anonymously and allow community workers to inform people on where to find additional support.

Future research could focus on understanding whether there are causal links between climate change and the coping strategies employed by individuals and communities. It is important to explore the consequences of using different coping strategies, particularly those that are considered high-barrier versus low-barrier, on health and adaptation capacity across various contexts. Additionally, future studies could investigate the direct associations between the use of specific coping strategies and how these associations might shift when dealing with climate change-related stress. This research should also consider whether the total amount of stress, rather than the nature of the stressor itself, plays a more significant role in influencing coping behaviors.

### Limitations

The study was conducted during the COVID-19 pandemic, which impacted our research in several ways. Firstly, training for the RAs had to take place online, and debriefings were conducted via phone, which may have impacted data quality. Data was collected within a short period of time to reduce health risks for RAs and participants, which prevented us from achieving prolonged engagement in the field. Secondly, safety measures during the interviews may have influenced rapport building with the participants. Furthermore, though RAs were advised to select an equal amount of women, most women we approached were not allowed to freely make decisions regarding participation in the interviews and often sought their partners’ permission. These cultural norms account for the uneven gender distribution. However, as women shared deep insights into their coping behavior when allowed to participate, their perspectives take up a similar space as men’s in the interviews.

## Conclusion

Psychological coping as a reaction to general stress gives insights into adaptation efforts, both regarding psychology and behavior. We show how barriers to coping can lead people to use more easily accessible strategies, such as resignation, which may be maladaptive when used frequently. Maladaptive coping can hinder adaptation through exhausting resources that may be necessary in the future. While maladaptive psychological coping such as resignation can lead to decreased motivation and efforts to adapt psychosocially, its behavioral impacts can also jeopardize adaptation by preventing people from taking action and learning. We discuss that environmental stress specifically seems not to shape coping in a particular way, but rather that the feeling of uncontrollability leads people to cope more passively, which is similar to findings from research on other stressful environments. As the negative impacts of climate change will be felt globally with more intensity and frequency, removing barriers to adaptive coping strategies will be essential for enabling successful adaptation efforts in the future.

## Supplementary Information

Below is the link to the electronic supplementary material.

Supplementary file1 (DOCX 28 KB)

## Data Availability

Original data is not openly available to ensure participants’ privacy.
