# Supplementary material for: “What Can We Do?”: A Psychological Perspective on (Mal)Adaptive Coping Strategies and Barriers to Coping in an Area of Severe Climate Vulnerability in Bangladesh
Source: Int J Behav Med. 2024 Nov 26;33(3):461–75. doi: 10.1007/s12529-024-10329-8 (PMC13342328; doi:10.1007/s12529-024-10329-8)
Supplement: Supplementary file 1 — Supplementary file1 (DOCX 28 KB) [file 12529_2024_10329_MOESM1_ESM.docx]

**Supplemental material**

**Quotes**

| Quote number | Example Quote | Associated Stressor |
| --- | --- | --- |
| Q1 | When it feels worse, I lock the door of the room and lie down. Sometimes I cry, I keep my pain pressed inside my chest. (Man, 20) |  |
| Q2 | When I feel bad, I lie down, I do some work; then when I feel like crying, I cry. (Woman, 19) |  |
| Q3 | If I talk about my pain, what's the use of it, husband is ill he is totally ill, now I feel sad suddenly.[…] I cry to Allah and stay like that, what can I do…. (Woman, 25) | Illness |
| Q4 | I think about these things, there is no shelter house, no madrasa, no big building, I cannot sleep in peace thinking of all these [issues]. (Man, 35) | Poor Infrastructure |
| Q5 | It is scary. There is no guarantee, now the river is silent, not sure it will be angry at any time. Yes, [I have trouble sleeping]. Don’t you see my face is full of pimples? (Woman, 35) | Floods |
| Q6 | This year I had heart problems for thinking about business. (Man, 40) | Financial scarcity |
| Q7 | I want them [children] to get educated and become a good human; if they get educated then we can get them married in a good place. […] For this I fall sick, for this tension I fall sick. I always have this tension. (Woman, 25) | Societal conventions |
| Q8 | It [domestic violence] happens. […] It can be seen when husband’s income is low, or he has not got a job today. He has children at home, he has a wife. They must be fed and he is tense. When the wife says something, there starts a fight. […] Such problems happen in every house. (Woman, 31) | Financial scarcity |
| Q9 | Why will he scold me, I scold him [husband] instead. Why do you stay at home? Why, for your friends? For friends, family life gets ruined. They [husband’s friends] used him for money. That’s why we quarrel at home.  (Woman, 19) | Financial scarcity |
| Q10 | Sister, people expect from small to big. […] I sleep on the ground, dreaming of the car will benefit me? […] There was hope. I didn't say that, if I had such an income, if I could find a way to walk now, then I would dream that I would buy a land.[…] I do not have any such situation. What is the benefit of dreaming?! (Woman, 30) | Financial scarcity |
| Q11 | My parents made me understand that we are born in a poor family, Allah gives everything what else can you do? What do you guys do over Allah? If you act against Allah then nothing will happen, we will only have death. (Woman, 18) | Financial scarcity |
| Q12 | Because of the waves […] [river] erosion happens. […] Also due to weather, when there is a storm the air pressure is higher, for that it [river erosion] can happen. This is a climate change issue. Because it is in our country, it is our responsibility. […] It [climate change] is related to all over the world, if other countries can control the weather, Bangladesh should also do it. (Man, 30) | Climate change |
| Q13 | Sometimes I feel depressed for my business, if it doesn't work today, it will work out tomorrow, I have hope. (Man, 50) | Financial scarcity |
| Q14 | Then if I can't [realize my plan], I'll go the other way. Business or something else. I want to grow based on my willpower. If I slip down, I will rise by myself. (Man, 18) |  |
| Q15 | *People got their trees broken or it takes away the houses and shops, destroy crops, cattle dies, […] What can I do with this! If god wants to kill us, what can we do? (Man, 37*) | *Cyclones* |
| Q16 | If the leader of a region is dishonest it will cause problems. If you are assigned as my protector and you are the one harming me, we have nothing we can do. (Man, 30) | Politics |
| Q17 | I don’t have any future now, now my future is Allah […] I don’t want extra days to live. Now I have got old, what’s the use of surviving now? If I die suddenly then that’s my success and if I die suffering then who will look after me? There is no one except for Allah. […] I am not that important for this family. (Woman, 60) |  |
| Q18 | The illness I have in my body, what will I tell you about it, I have many illnesses dear, what is the use of talking about them. Like I told you, I am not able to eat rice; my gum bleeds. […] for this I have grown thin for the loss of blood […] …what can I do. (Woman, 60) | Illness |
| Q19 | There were neighbours and we used to have fun […]and then I used to feel like sharing with them. They used to give me hope. […] I don’t go to anyone from our house. I go to the girl who taught me stitching; her mother is like a sister, I go to them; then there is this aunt, she calms me down. (Woman, 18) |  |
| Q20 | Yes we used to live like one family, now we do not see anyone, I feel so bad for them, I call via phone with some of them, sometime we meet after a month, we sit together and drink tea, spent some money for each other, we really miss each other, some even cry for that […]the people living beside me now, we sometimes give and take some money from each other, […] that is how we live and survive together. (Man, 35) | Social disruption |
| Q21 | I am in a lot of cost (crying a lot). It is not right to say these things, for us. Don’t ask my heart’s words. I am saying to you all, but it is not right to say. (Man, 78) | Financial scarcity |
| Q22 | No, I don’t want to [share sorrows], because people will call me bad. Why would I share with others, I keep it to myself. (Woman, 18) |  |
| Q23 | I lay down [when the blood pressure is high]. When I see that it is worse, I consult a doctor and measure my blood pressure to tale medicine. (Woman, 30) | Illness |
| Q24 | It’s like, if I need anything, beside our house there is mami (Aunty. She calls her neighbour aunty), people living in this are like our relatives; we all stay together, […] that day I came here to mami. Told her that we don’t have onions, took few onions or if I need salt I take, oil I take it from her. And when we bring those for our house, we give them back. This is how we work. (Woman, 25) |  |
| Q25 | What will be the benefit if I protest alone, sister. This will have to be done by 10 more people. […] They have the intelligence to walk with the members [local politicians]. But I don't have that intelligence. There is no power. There is no money. […] I went in front of the Member when I was sick. […] Then I actually registered [for relief] . Sister, but then rice came twice more but my name was not registered […]. If the member does not take my information, then the mind will not allow to go twice! Even if they die at home without eating, they will not want to go. (Woman, 30) | Politics |
| Q26 | If we raise our hands for help then where will respect and values go? We don’t ask for help or go to them. (Woman, 60) |  |

## **Interview guide**

Good morning/afternoon,
Thank you for meeting with me today.

I have asked you to meet with me in the hopes of learning more about your experience and perceptions related to your health and migration. We are interested to learn any of your thoughts regarding this. Some of the questions I will ask you may not want to answer and that is fine. Remember that your participation is completely voluntary. Also please keep in mind that there are no right or wrong answers, I am interested in anything you can share with me.

As we went over in the consent, we will be taking notes and also recording our conversations so that we can accurately capture and report your views. Your names will not be recorded.

Your comments will be combined with those from other meetings with residents from this area and residents from Dhaka.

Just as a reminder our discussion will probably last around 60 minutes. Some of the questions I will ask you may not want to answer and that is fine.
Do you have any questions before we begin? May I start the recording? *[Start recording]*

| 1. Please tell me something about yourself   probe: profession/education, status in community/ family, history, health, financial status, migration experience |
| --- |
| 1. Please tell me about a normal day here in Bhola   probe: work, family, community, social activities |
| 1. Please tell me something about this area.   probe: changes in weather, population, security, personal views like/dislike, health care access, poverty, culture/religion, identity/ place attachment, community/family structure, development, jobs (new kinds of jobs, amount) |
| 1. What do you think are problems in this area? |
| 1. How do you think these issues effect men?   probe: finding work, emotional health, physical health, solutions |
| 1. How do you think these issues effect women?   probe: emotional health, physical health (maternity, pregnancy), family structure (head of household), violence (sexual, domestic), work/education, health care access, solutions |
| 1. How do you think these issues effect children?   probe: health, emotional health, education, work, safety, solutions |
| 1. What do you think will happen in this area in the future?   probe: development and its consequences (for people, environment) |
| 1. What do you think about migration from here?   probe: where people go, why, do they come back |
| 1. How do you think migration has affected this area?   probe: infrastructure, jobs, community/family structure, health care access, physical health/emotional health of residents, work distribution, remittances, neglected relatives, more work/pressure on staying people |
| 1. How do you think migration from here has affected you personally/ your family?   probe: physical and emotional health, health care access behaviour, social life change, change in work burden |
| 1. What do you think are the alternatives to migration? |
| 1. How can people from here achieve these alternatives? |
| 1. What are your plans for the future? |
| 1. Where do you think your family/community will be in the future?   probe: immediate future and long term |
| 1. What are your concerns for the future here? |
| 1. What kind of life do you think the children from this area will have?   probe: will they be here? Education / livelihoods |
| 1. What do you think of the health care in this area? |
| 1. How do you access health care? Where do you go? |
| 1. What kind of illnesses have you experienced? |
| 1. Have they changed over the past years and, if yes, why? |
| 1. If you could speak directly to a government official, what would you say to them?   probe: personal support, area support: infrastructure / facilities / government interventions |
| 1. Is there something I should have asked you that you would like to add? |

Probes refining:

Emotional/mental health: How do people feel? Depressed, anxious, angry, relieved, happy, sad, vulnerable, active, stressed, tensed…

If they share about challenging emotions > probe: what do they do to handle them? How do they cope with them? What helps them to relax or relief their stress?

Community/ family structure: Which role do people play? Who decides what? What expectations do people face? Who is head of household? What is percieved as good/bad in the community?

Health care access: Ability to access health care, behaviour/opinions on health care
